# Indonesian Mangrove *Sonneratia caseolaris* Leaves Ethanol Extract Is a Potential Super Antioxidant and Anti Methicillin-Resistant *Staphylococcus aureus* Drug

**DOI:** 10.3390/molecules27238369

**Published:** 2022-11-30

**Authors:** Kholis Abdurachim Audah, Jufendi Ettin, Jason Darmadi, Norma Nur Azizah, Amalda Siti Anisa, Tedi Dwi Fauzi Hermawan, Conny Riana Tjampakasari, Rudi Heryanto, Intan Safinar Ismail, Irmanida Batubara

**Affiliations:** 1 Department of Biomedical Engineering, Swiss German University, Tangerang 15143, Indonesia; 2 Directorate of Academic Research and Community Service, Swiss German University, Tangerang 15143, Indonesia; 3 Drug Development Research Center, Indonesia Medical Education and Research Institute, University of Indonesia, Jakarta 10430, Indonesia; 4 Department of Clinical Microbiology, Faculty of Medicine, University of Indonesia, Jakarta 10430, Indonesia; 5 Tropical Biopharmaca Research Center, IPB University, Bogor 16128, Indonesia; 6 Department of Chemistry, IPB University, Bogor 16680, Indonesia; 7 Institute of Bioscience, Universiti Putra Malaysia, Serdang 43400, Malaysia

**Keywords:** MRSA, mangrove, *Sonneratia caseolaris*, bioactive compound, disk diffusion, antibacterial activity, super-antioxidant, phytochemical

## Abstract

Methicillin-resistant *Staphylococcus aureus* (MRSA) is an *S. aureus* strain that has developed resistance against ß-lactam antibiotics, resulting in a scarcity of a potent cure for treating Staphylococcus infections. In this study, the anti-MRSA and antioxidant activity of the Indonesian mangrove species *Sonneratia caseolaris*, *Avicennia marina*, *Rhizophora mucronata*, and *Rhizophora apiculata* were studied. Disk diffusion, DPPH, a brine shrimp lethality test, and total phenolic and flavonoid assays were conducted. Results showed that among the tested mangroves, ethanol solvent-based *S. caseolaris* leaves extract had the highest antioxidant and anti-MRSA activities. An antioxidant activity assay showed comparable activity when compared to ascorbic acid, with an IC50 value of 4.2499 ± 3.0506 ppm and 5.2456 ± 0.5937 ppm, respectively, classifying the extract as a super-antioxidant. Moreover, *S. caseolaris* leaves extract showed the highest content of strongly associated antioxidative and antibacterial polyphenols, with 12.4% consisting of nontoxic flavonoids with the minimum inhibitory concentration of the ethanol-based *S. caseolaris* leaves extract being approximately 5000 ppm. LC-MS/MS results showed that phenolic compounds such as azelaic acid and aspirin were found, as well as flavonoid glucosides such as isovitexin and quercitrin. This strongly suggested that these compounds greatly contributed to antibacterial and antioxidant activity. Further research is needed to elucidate the interaction of the main compounds in *S. caseolaris* leaves extract in order to confirm their potential either as single or two or more compounds that synergistically function as a nontoxic antioxidant and antibacterial against MRSA.

## 1. Introduction

Antibiotics have revolutionized not only the medical field but also a huge array of sectors ever since their discovery and industrialization. Through their ability to selectively target the microbes while simultaneously not harming the host, antibiotics were regarded as a perfect drug that could potentially cure any infections. In principle, antibiotics selectively target microbial processes such as replication and metabolism [1,2]. Unfortunately, overuse of antibiotics promotes the emergence of antibiotic-resistant bacterial strains [3]. Surviving bacteria acquired antibiotic-resistance-associated gene mutation, which allows regulation and expression of antibiotic-interfering proteins. Bacteria are more prone to developing mutations over multiple generations as a side effect of binary fission [4]. This phenomenon leads to the collection of multiple antibiotic resistance within one species [5]. One example of mutation response against the environment is in the gene *flhF* in polar flagellated bacillus, where the gene encodes FlhF for regulating the assembly of various surface organelles including flagella. Multiple point mutation and deletion have been reported in *Pseudomonas aeruginosa* as a defense mechanism to survive more viscous liquid and for higher infectivity in humans [6].

Methicillin-resistant *Staphylococcus aureus* (MRSA) is a strain of Gram-positive *S. aureus* which has been causing serious health problems. The strain has become genetically distinct from its root species and has also acquired a multidrug resistance trait. Generally, *S. aureus* lives on the skin’s surface and does not pose a major threat but can lead to a very contagious infection if the bacteria manage to invade the bloodstream. Because MRSA strains acquired *mecA* genes which encode a PBP2a enzyme, they are resistant to nearly all β-lactam antibiotics [7]. This gave MRSA the ability to be resistant to most generic antibiotics used for treating staphylococcus infections, such as penicillin and cephalosporins.

The majority of available drugs from the past few decades are derived from natural products or their derivatives. These compounds are found and extractable from microbes, plants, or other living beings in the form of secondary metabolites as biosimilars [8]. In plants, phytochemicals are synthesized for signaling and communicating with symbiotically related species as well as for self-preservation against external factors ranging from oxidative stresses and predation. As a result, some phytochemicals may develop antioxidant and or antimicrobial activity [9]. Some antioxidative flavonoids and tannins are able to exhibit high levels of antibacterial activity and may be able to inhibit MRSA growth due to their phenolic structure, which contains moieties that are able to scavenge radicals [10]. Studies have also shown that phytochemical bioactivity is highly influenced by ecological stresses [11]. This is especially true for marine plants that live in high salinity and are exposed to more microorganisms, thus increasing the amount and potency of each metabolite [12]. Hence, it is apparent that plants that are able to withstand harsh environments might be a good potential for an alternative antibiotic source, such as mangroves.

Mangroves are salt-tolerant plants that grow in coastal and brackish marshes. They are capable of anchoring their roots under marine shores and serve as a prominent lynchpin for various complex marine and terrestrial ecosystems [13]. Because they have adapted to grow surrounded by various environmental stresses, mangroves are found to produce protective bioactive phytochemicals and are thus known to be a promising source for extracting such compounds [12]. There have been studies documenting the medicinal benefits of the extracted secondary metabolites of local mangroves species due to their biological activity. Performing research on local mangrove species is also beneficial as there is historical, agricultural, and ethnomedicinal significance for the inhabitants as well as the environment surrounding each mangrove forest [14]. Studies have shown that the extracted mangrove leaves could be used as a source for the naturally derived antibacterial drug as they contain high traces of phenolic compounds, particularly phenolic acids and flavonoid derivatives [15]. Several mangrove species of Indonesian locale *Sonneratia caseolaris*, *Avicennia marina*, *Rhizophora mucronata*, and *Rhizophora apiculata* were proposed to be the specimens of this research as a continuation of a preliminary study performed by Audah et al. [16], where the leaves extract of the aforementioned species yielded strong antimicrobial activity. It is hoped that the local mangrove species yield high amounts of antioxidative polyphenols that are highly antimicrobial against the MRSA strain in order to increase the bioprospecting of local mangrove species for drug discovery [13]. This study is very important in providing viable alternatives in combating the emergence of multidrug-resistant (MDR)-conferring bacteria and microbes in general.

## 2. Materials and Methods

All experimental research and field studies on plants complied with relevant institutional, local, national, and international rules and guidelines. Details of the samples used in this study were written in our previously published works [16,17]. The details include location, identification of plant materials, personnel, and the authority bodies involved.

### 2.1. Leaves Simplicia Extraction and Solvent Determination

Simplicial leaves of *S. caseolaris*, *A. marina*, *R. mucronata*, and *R. apiculata* were obtained from Kawasan Konservasi Mangrove, Propinsi Lampung, Indonesia. They were then extracted by means of maceration using different solvents. The extracts were evaporated using locally-bought solvents with different polarities: ethanol, ethyl acetate, hexane, and water. Ethanol was chosen as the main solvent for the *S. caseolaris* leaves extraction. Water was used for the extraction of *A. marina* leaves, *R. mucronata* leaves, and *R. apiculata* leaves. The deliberation of each solvent chosen was based on the highest simplicial extraction mass yield and preliminary tests on nontoxicity using a brine shrimp lethality assay (BSLT) [16].

### 2.2. Quantitative and Qualitative Phytochemical Analysis

Simplicial leaves were phytochemically analyzed qualitatively by Mayer’s test, Wagner’s test, and Dragendroff’s test for alkaloid detection. Other tests include the froth test for saponin detection, the phenol Ferric Chloride test for tannin detection, as well as Liebermann Buchard’s and Salkowski’s test for steroids-triterpenoids detection [16,18]. The hydroquinone content was tested by an alkaline reagent test [19]. Quantitative tests were also performed for measuring the total phenolic content and flavonoid content of the extracts.

#### 2.2.1. Total Phenolic Content (TPC) Determination

The method was adapted from Audah et al. with several modifications in the procedure [16]. The extract paste was dissolved using 75% ethanol (Merck, Germany). The extracts were mixed with Na_2_CO_3_ 2% (Sigma Aldrich, Saint Louis, MO, USA), and locally bought Folin-C. Reagent 10% with a mixture ratio of 1:2:1 for each chemical. The mixtures were incubated for 30 min in darkness at room temperature. Mixtures were then read at 765 nm wavelength using a Cary 60 UV light-visible (UV-vis) spectrophotometer (Agilent Technologies, Santa Clara, CA, USA). TPC concentration of extracts was calculated using a standard calibration curve made from gallic acid (Sigma Aldrich, Saint Louis, MO, USA) from concentrations 0–100 ppm.

#### 2.2.2. Flavonoid Content (FC) Determination

The method was adapted from Audah et al. with several modifications in the procedure [16]. The extract paste dissolved in 75% ethanol was mixed with 75% ethanol, 20% of locally bought AlCl_3_, 2 M of CH_3_COOK (BDH Middle East, Dubai), and aquadest with a volume ratio of 5:15:1:1:28. The mixtures were incubated for 30 min in darkness at room temperature and were read at 440 nm UV-vis. The FC concentration of extracts was calculated using standard quercetin (Sigma Aldrich, Saint Louis, MO, USA) at 0–200 ppm.

### 2.3. Antioxidant Activity Determination by DPPH Radical Scavenging Assay

The antioxidant assay was performed as reported by Jadid et al., with several modifications in the procedure [18]. Different solvent-based extracts and ascorbic acid (Merck, Germany) dissolved in 75% ethanol at concentrations of 0–100 ppm were mixed with DPPH (Sisco Research Laboratories, Mumbai, India) 100 ppm (1:1). The mixtures were incubated for 30 min in a dark chamber at 25 °C and read at 517 nm UV-vis with both DPPH IC_50_ made from standard control calibration curve DPPH at a concentration of 0–100 ppm. IC50 of ascorbic acid and extracts were then compared with each other.

### 2.4. Brine Shrimp Lethality Test (BSLT) for Extract Toxicity Assay

The experiment was performed according to the methods reported by Dosumu et al., with several modifications in the procedure [20]. The test was done in triplicates, where extract pastes dissolved with seawater at concentrations of 0–1500 ppm were mixed with seawater (1:1) filled with 10 *A. salina* nauplii hatched 24 h before the experiment in a CO_2_ aerator. Mixtures were incubated for 24 h at 25 °C and the BSLT LC_50_ of the extract was calculated using probit analysis, with positive control 50% ethanol and negative control seawater solution treated to the same amount of brine shrimps.

### 2.5. Agar Disk-Diffusion Assay for Antibacterial Activity Determination against MRSA Strain

The experiment was performed based on the methods of a Kirby-Bauer Disk Diffusion Susceptibility Test reported by Hudzicki et al. [21]. Each test was carried out in five replicates for reproducibility. The MRSA bacteria strain was adjusted to 1 × 10^8^ CFU/mL or 0.5 McFarland’s standard and was further inoculated onto the entire surface of a Mueller-Hinton agar (MHA) plate. An even lawn was made in MHA using a sterile swab [22]. Ethanol extracts dissolved in 75% ethanol from stock solution were prepared for concentrations of 1250–20,000 ppm. Then, 6 mm filter paper disks were impregnated with 20 μL diluted extracts and dried out in a sterile condition. The impregnated disks were then placed on top of the surface of each MHA plate to be incubated for 24 h at 37 °C. The diameter of the zone of inhibition (ZOI) formed between the disks was measured, with a larger diameter meaning higher susceptibility of MRSA against the extracts. Vancomycin (VA5) antibiotic disks were used as positive control by placing the disk in the middle of the agar plate.

### 2.6. Liquid Chromatography-Mass Spectroscopy (LC-MS) for Extract Compound Characterization

The extracted leaves paste of *S. caseolaris* from the ethanol solvent was dissolved into 1 mL methanol. The mixture was injected into the LC column at a flow rate of 0.2 mL/min at 30 °C. The system of LC-MS/MS was UHPLC Vanquish Tandem Q Exactive Plus Orbitrap HRMS from Thermo Fisher Scientific, Waltham, MA, USA. The ionisation mode on the MS system used was negative ionisation. The scan mode used was a full scan from 100–500 *m/z*. The UPLC column used was Accucore C18, 100 × 2.1 mm, 1.5 µm (Thermo Fisher Scientific, Waltham, MA, USA). The eluent used was H_2_O + 0.1% Formic acid (A) and acetonitrile + 0.1% formic acid (B). The results of the analysis were displayed using a mass chromatogram.

### 2.7. Statistical Analysis

All values were expressed as mean ± standard deviation, with all samples being made at least in triplicates. Statistical analysis was carried out using probit analysis for LC_50_, performed in Microsoft Excel 2013. Linear regression was also performed in Microsoft Excel 2013, with sigmoidal fitting performed in Origin 2021 Academic (License 95E from KIT institution) using Hill’s model equation as a basis.

## 3. Results and Discussion

### 3.1. Dried Leaves Simplicial from Local Mangrove Species Phytochemical Extraction and Characterization

Dried simplicial powder from the leaves of *Sonneratia caseolaris*, *Avicennia marina*, *Rhizophora mucronata*, and *Rhizophora apiculata* was analyzed qualitatively using various phytochemical tests, with the results shown in Table 1. In all instances, steroids were found, and high traces of tannins were also measured, confirming the presence of phenolics in leaves. Flavonoids and saponins were also detected in some species, albeit in lower concentrations [16].

Based on Table 2, all species except *S. caseolaris* leaves yielded the most mass percentage when extracted using water, whereas *S. caseolaris* leaves yielded the highest when using ethanol. All of the highest mass-yielding extracts, which will be referred to further in this article as the extracts, produced varying levels of toxicity when tested with the brine shrimp lethality test (BSLT). The concentration needed for ethanol-based *S. caseolaris* leaves extract to reach 50% lethality on *Artemia salina* brine shrimp (LC_50_) was higher than 1000 ppm, noted to be nontoxic by the toxicity criterion published by Meyer et al. [23]; while the others were deemed as slightly toxic as they were near the 1000 ppm threshold. All of the highlighted extract solvents (Table 2) were used for further assays to determine their phytochemical activity levels.

It was suggested that the less toxic and more biologically active compounds were more likely to be extracted using a highly polar solvent and were in abundance in the main extracts, such as the case in ethanol-based *S. caseolaris* leaves. This was supported as the detected phytochemicals in most of the dried powder leaves were mostly polyphenols (tannins and flavonoids) as well as steroid-based chemicals such as saponins, which are mostly attributed to life-supporting bioactivity such as antioxidant activity [24,25]. Due to the polarity of ethanol, phytochemicals classified as phenolics as well as steroids have more affinity towards alcohol-based solvents and water to a lesser extent [19]. However, some antioxidative phytochemicals including phenolic compounds are able to induce acute toxicity in humans [20,26].

### 3.2. Toxicity of Various Local Mangrove Species Leaves Extracts

The toxicity of extracts was assayed using the brine shrimp lethality test (BSLT), with brine shrimp *Artemia salina* used as an indicator species for lethality by incubating for 24 h. Based on the toxicity criterion, all extracts demonstrated non-lethality with LC_50_ of >1500 ppm, which might indicate the degradation of phytochemicals over time [23]. Antioxidants are prone to instability after multiple instances of quenching free radicals due to the limited electrons in their structure as well as other external factors which influence the rate of degradation such as shelf life, storage temperature, and humidity [17,27,28]. This suggested that the phytochemicals contained in the extract had degraded to the point where they were non-toxic anymore for the model animals.

Solvents also play a large role in inducing toxicity in experiments and extracts. In previous experiments [16,29,30], extracts were diluted in Tween80, while the ones performed in this experiment were diluted in only seawater. As a surfactant, Tween80 is more toxic compared to other natural organic solvents (used in maceration in Table 2) because of its ability to denature proteins and inhibit some biological processes [31,32].

Some nauplii in the experiment were found to move faster and more energetically compared to before incubation in the extract. Even after another incubation period of 24 h, the brine shrimp specimens were still viable. It was hypothesized that mangrove extracts provided dietary nutrients to brine shrimps, as some mangrove plants are used as a food source for humans. In previous studies, human adipose-derived stem cells incubated in several ethyl acetate-based local mangrove extracts had increased cell growth to over 100% viability while still being able to exhibit selective cytotoxicity towards various cancer cell lines [17,33].

### 3.3. Antioxidant Activity of Phytochemicals in Various Local Mangrove Species Leaves Extract

**Figure 1:**
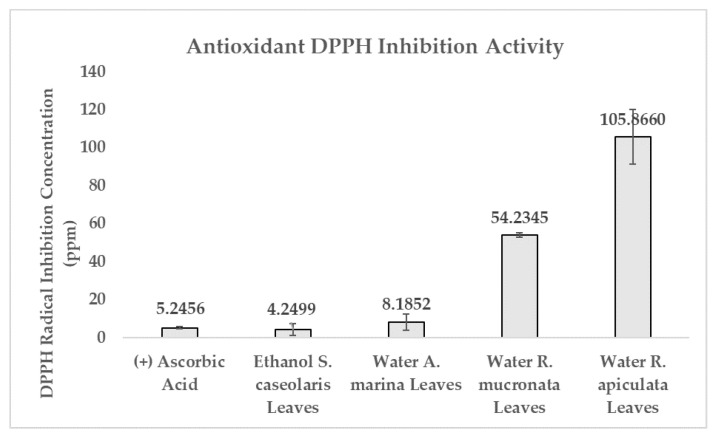
Comparison of DPPH IC_50_ between controlled ascorbic acid and each mangrove species extract. Result values and error bars were plotted in the form of mean ± standard deviation, respectively.

DPPH radical scavenging activity was performed to determine whether the extracts contained antioxidant activity by comparing them against controlled ascorbic acid. Ascorbic acid is capable of rapid radical oxygen species (ROS) elimination, formation inhibition, as well as preventing oxidation pathways [34]. Antioxidant activity was calculated in terms of concentration where DPPH inhibition reached 50% (DPPH IC_50_). Water-based *A. marina* leaves extract was able to inhibit DPPH radicals in an equivalent concentration and efficacy as ascorbic acid. Ethanol-based *S. caseolaris* leaves extract was able to inhibit in a slightly smaller concentration compared to ascorbic acid with the value of 4.2499 ± 3.0506 and 5.2456 ± 0.5937 ppm, respectively, as shown in Figure 1 and Supplementary Table S1. Using the criterion of antioxidant potency performed by Phongpaicit et al., water-based *R. mucronata* and *R. apiculata* leaves extracts were considered to contain intermediate and weak antioxidant activity, while ethanol-based *S. caseolaris* leaves and water-based *A. marina* leaves extracts were considered as having very strong antioxidant activity [18,35]. Due to the water-based *A. marina* leaves extract having comparable inhibition efficacy of ascorbic acid while ethanol-based *S. caseolaris* leaves extract outperformed ascorbic acid; both extracts, and therefore their species, could be considered as having super-antioxidant potential.

**Figure 2:**
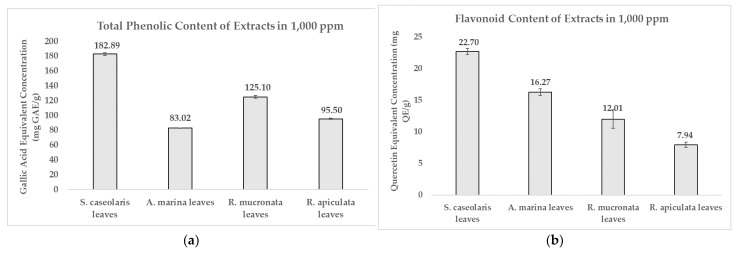
Comparison of: (**a**) Total Phenolic Content; (**b**) Flavonoid Content of various local mangrove species extracts dissolved in 1000 ppm. Result values and error bars were plotted in the form of mean ± standard deviation, respectively.

It was found (Figure 2a) that the ethanol-based *S. caseolaris* leaves extract contained the most abundant phenolic content at 182.89 ± 1.76 mg Gallic Acid Equivalent (GAE)/g, with flavonoids making up to 12.4% of the compounds at a concentration of 22.70 ± 0.48 mg Quercetin Equivalent (QE)/g (Figure 2b). However, the highest flavonoid percentage was achieved by the water-based *A. marina* leaves extract at 19.6% of total phenolics (Supplementary Table S1). Phenolic contents could be grouped into three categories based on how concentrated they were: high-concentration phenolic content at >70 mg GAE/g, moderate-concentration at 10–70 mg GAE/g, and low-concentration at <10 mg GAE/g [33]. Based on this criterion, all of the extracts could be categorized as highly phenolic. The quantified phenolics and flavonoid results for each solvent extract clearly matched the preliminary qualitative phytochemical screening in Table 1, where the dried leaves simplicial had yet to be extracted in different solvents.

Tannins and flavonoids are phytochemicals classified as phenolic compounds due to the fact they contain phenols or derivatives in their core structure. The presence of phenols as well as at least one other moiety such as hydroxyls results in most tannins and flavonoids being able to scavenge free radicals [9,36]. After donating proton ions [H+] to ionically unstable compounds or free radicals, phenolic phytochemicals could regain their charge balance from the resonating aromatic structure, and may still donate their protons to reduce the oxidative imbalance in other radical-containing compounds. Besides hydroxyls and glycosides, other moieties and structural formations are also able contribute greatly to antioxidant activity by overall polarity and hydrogen bonds [37]. Some tannins and similarly-structured flavonoid derivatives (condensed forms of anthocyanins, flavan-3-ols, and catechins) are able to scavenge radicals due to their high polymerization capability to form more stable structures [36,38]. The possession of multiple antioxidant-associated structures as well as the aforementioned mechanisms are why most flavonoids and tannins are considered highly or moderately-active antioxidants [39].

Secondary metabolite formations, especially antioxidant phenolics, are mostly attributed to the evolutionary product of the plants’ natural defense mechanisms against the harsh environment and predation [40,41]. Most mangroves are located in brackish waters and swamplands where salinity and high concentration of microorganism niches reside [12,13]. It was believed that mangroves are able to regulate salt and ionic uptake by shedding their old leaves, which are used to store excess salt, generating salt glands on their leaves and stems, or ultrafiltration through their roots [42]. The mangrove species used in this study were located in a local estuary and near the shorelines, which explains the rich antioxidant activity as well as the phenolic compounds found in the very strong antioxidative extracts. While the only species in this study known to shed leaves and expel salts from their leaves in wide salinity fluctuations are *A. marina*, which was the most salt-tolerant out of the four species, *S. caseolaris* was also able to produce highly antioxidant phenolic phytochemicals. This indicates other factors can play a role in phytochemical emergence, which could be an interesting follow-up study to understand the difference between extrinsic and intrinsic factors throughout the ethnomedicine of certain same-mangrove species grown in a different locale.

### 3.4. Antibacterial Activity of Various Local Mangrove Species Leaves Extract against MRSA Strain

**Figure 3:**
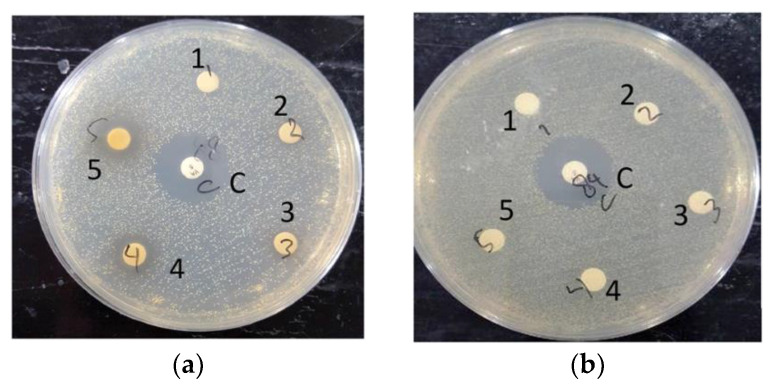

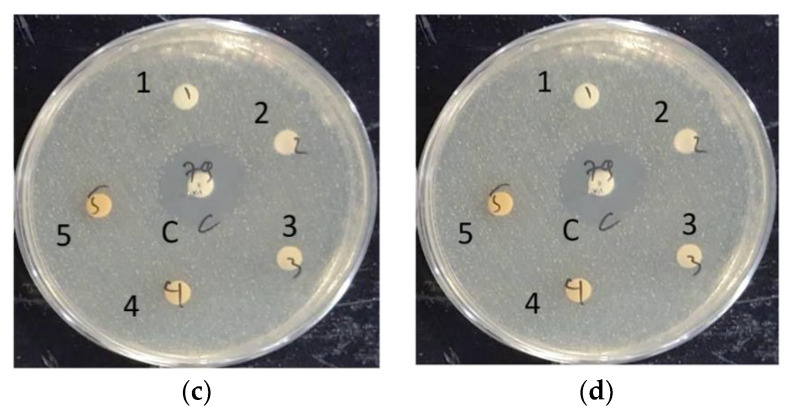
Documented run for: (**a**) ethanol-based *S. caseolaris* leaves extract; (**b**) water-based *A. marina* leaves extract; (**c**) water-based *R. mucronata* leaves extract; (**d**) water-based *R. apiculata* leaves extract on LB agar medium grown with MRSA strain. The middle disk labeled C is positive control Vancomycin, while the numbered labels were concentrations of diluted extracts. Label number **1** is the smallest extract concentration at 1250 ppm, number **2** is 2500 ppm, number **3** is 5000 ppm, number **4** is 10,000 ppm, and number **5** is the largest concentration at 20,000 ppm.

A preliminary test was conducted using a non-sterilized 6 mm diameter 0.22 μm filter paper disk, impregnated with extracts. When the non-sterile extract-impregnated disks were tested against MRSA, the disks were found to be heavily contaminated. This was also the case for the sterilized disks, with all extracts except the ethanol-based *S. caseolaris* leaves extract. The *S. caseolaris* leaves extract did not show any inhibition around the disk vicinity, with MRSA colonies growing very near to the impregnated disks (Figure 3a–d). This did not fall in line with what had been reported in an earlier study where the ethanol-based *S. caseolaris* leaves extract did not show significant *S. aureus* inhibition. In the aforementioned study, extracts in different solvents were tested against the Gram-negative bacteria *Escherichia coli*, *Pasteurella mosselii,* as well as Gram-positive bacteria *Propioniumbacterium acnes*, *S. aureus,* and *Rhodococcus equi*. The *S. caseolaris* leaves extract was reported to be able to inhibit *P. acne* with a zone of inhibition (ZOI) diameter of around 8 mm and 7 mm for ethanol- and water-based extracts, respectively. A water-based *A. marina* leaves extract was reported to inhibit *S. aureus* growth at around 10.7 mm. Both water-based *R. mucronata* and *R. apiculata* leaves extracts were also documented to inhibit *S. aureus* growth at ZOI 7 mm and 8 mm respectively [16].

**Figure 4:**
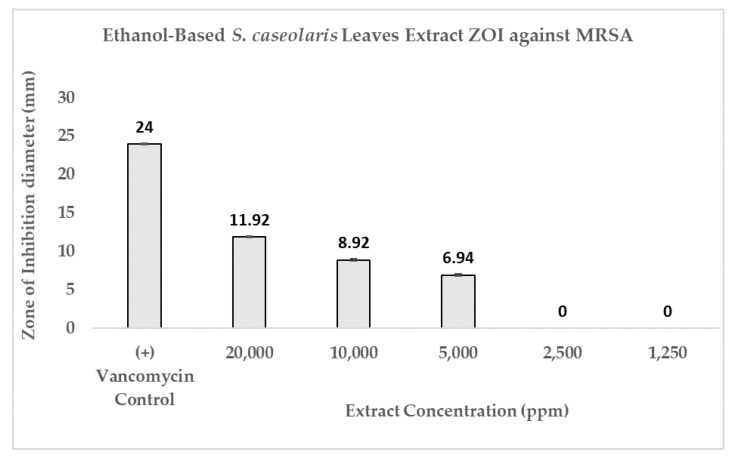
Antibacterial activity of ethanol-based *S. caseolaris* leaves extract against MRSA strain. Positive control was made from Vancomycin (5 μg), yielding a ZOI diameter of 24 ± 0.1 mm.

The disks were impregnated with 5000 ppm (3) until 20,000 ppm (5) *S. caseolaris* extracts were able to inhibit the growth of the MRSA strain, with a diluted extract of 20,000 ppm capable of inhibiting around half the range of control antibiotic Vancomycin. While visualized extracts of 1250 ppm (1) and 2500 ppm (2) (Figure 3a and Figure 4) contained small ZOI, indicating a very faint indication of MRSA growth inhibition, they were deemed to be non-significant due to irregularity in ZOI shape. Hence, the hypothetical minimum inhibitory concentration (MIC) of the ethanol-based *S. caseolaris* leaves extract was determined to be at around the range of 5000 ppm, or at 5000 mg/L where the least inhibition activity was seen.

**Figure 5:**
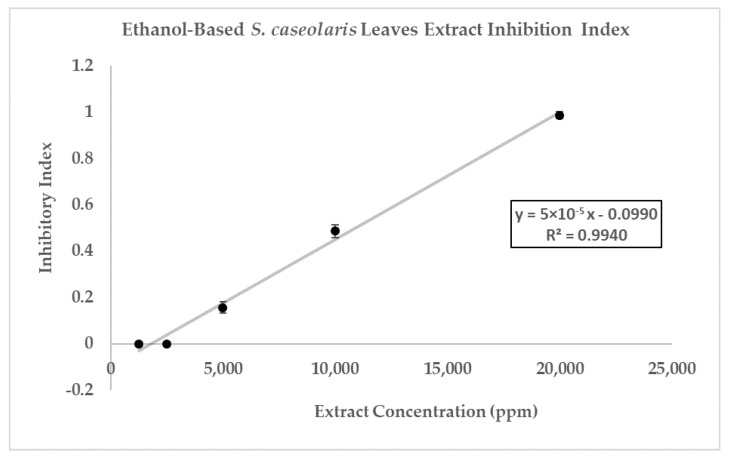
Inhibition index of ethanol-based *S. caseolaris* leaves extract against MRSA strain. Inhibition index of positive control Vancomycin (5 μg) was determined to be 3.0, using a calculation from its ZOI diameter.

The inhibitory index of each concentration of the *S. caseolaris* extract was also calculated and compared against positive control Vancomycin as well as fitted to linear regression to predict the inhibitory index range of the extract (Figure 5). While the inhibitory index increased proportionally, the concentrations used to reach the highest index was very high, at 20,000 ppm (200 mg/mL extract paste diluted in 75% ethanol) or 2% extract. Furthermore, the result could not even reach the inhibitory index of antibiotic control Vancomycin at 3.0. Using the breakpoint tables for of MICs and zone diameters from the European Committee on Antimicrobial Susceptibility Testing (EUCAST) Version 12.0 [43], the MIC breakdown for the Vancomycin-susceptible concentration of MRSA or *S. aureus* was determined to be ≤2 mg/L with the resistance to the strain accepted to be 2 mg/L. ZOI breakpoints were not published due to the unreliability of the disk diffusion assay to distinguish colonies containing a *vanA* glycopeptide-resistant gene. When the breakpoints were compared with the hypothetical MIC of the *S. caseloaris* extract, it was found that the crude extract did not fulfill the Vancomycin-susceptibility criterion for MRSA. The unusually high concentration of the sample extract might be attributed to the attenuation of antibacterial activity due to the crudeness of the ethanol-based *S. caseolaris* leaves extract, where a large number of different compounds interacted with one another.

There are various hypotheses surrounding the contradicting findings of the previous study [16]. One major reason is associated with the degradation of phytochemical compounds in the *A. marina*, *R. mucronata*, and *R. apiculate*, resulting in lower antibacterial activity, while the opposite occurs for the *S. caseolaris* extract. It was also suspected that not all of the anti-*S. aureus* compounds had degraded, and the substances left could not counter the multidrug-resistant (MDR)-aspect of MRSA, in which β-lactam-similar products were quenched by the PBP2a enzyme in MRSA [7]. Further studies on extract stability and extraction techniques are proven highly necessary to determine the true efficacy of all of the extracts.

Natural products and their sources exhibit antimicrobial activity to prevent pathogens caused by microbial infections. In this case, antimicrobial activity means actively inhibiting growth and other aspects of major microorganisms which cause infection, with more specific activities such as antibacterial, antifungal, and antiviral. There have been multiple instances of mangrove species producing antibacterial phytochemicals, with most reported compounds having a phenol functional group or derivatives [44].

There are reports of *S. caseolaris* found in different locations exhibiting anti-*S. aureus* as well as anti-MRSA. In one case, methanol-based *S. caseolaris* bark from Indian locale was able to exhibit significant antioxidative and antibacterial activity towards Gram-positive, Gram-negative, and yeast species [45]. Another study found that species grown in Thailand extracted in methanol were capable of inhibiting an MDR-conferring bacteria strain growth, such as the MRSA strain and extended-spectrum beta-lactamase-*E. coli* [46]. In both examples, the bioactivity was attributed to phenolics and antioxidant activity; where it has been investigated that the modes of action for several polyphenols correlate to inhibition of an array of enzymes in bacteria associated to metabolism pathways [15].

### 3.5. Phytochemical Compound Characterization by Liquid Chromatography-Mass Spectrometry (LC-MS) for Ethanol-Based S. caseolaris Leaves Extract Biological Activity-Compound Determination

**Figure 6:**
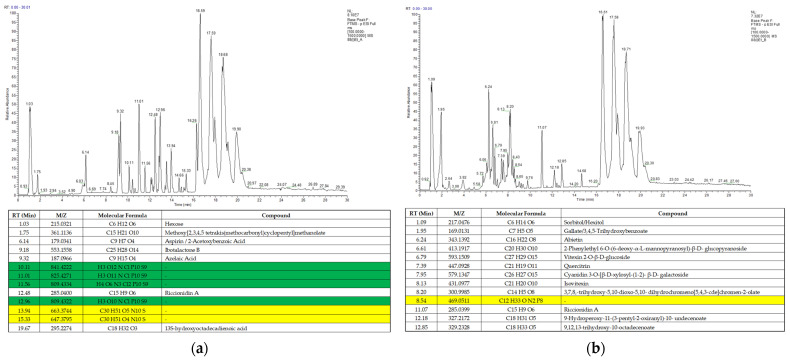
Chromatogram of ethanol-based solvent *S. caseolaris* leaves extract: (**a**) run A; (**b**) run B. Upper panels are chromatogram of LC-MS/MS data and lower panels are list of identified compounds.

In order to determine the presence of bioactive compounds and their interactions with one another, characterization using liquid chromatography-tandem mass spectrometry (LC-MS/MS) was conducted on the most biological activity-exhibiting crude extracts, and were the ethanolic *S. caseolaris* leaves extracts. Two separate runs were performed on the ethanol-based extracts, with an analysis of the chromatograms performed to determine antioxidant- and anti-MRSA-associated phytochemical compounds (Figure 6).

Analysis of the compound structure and formula in Figure 6a,b was carried out by comparing results with online chemical databases such PubChem, Human Metabolome Database (HMDB), ChemSpider, and MassBank. From the two runs, a total of 25 different compounds with known chemical formulas were found in the extracts, with most metabolites classified as phenolic compounds, while others were long-chained fatty acids and sugar macromolecules (Supplementary Table S1). Flavonoid-related compounds and other phenolic compounds similar in structure to tannins (in the form of ester gallates) or coumarins were found. One metabolite, called cyanidin 3-O-[β-D-xylosyl-(1-2)- β-D-galactoside], was found to be similar in structure to some monomeric precursors in condensed tannins [37]. Most of the phenolic metabolites such as cyanidin contained sugar moieties, which may be associated with the functions of compounds previously extracted from the leaves of *S. caseolaris* as signaling and allelopathy [47]. These flavonoid glycosides or glycoflavonoids could be an interesting finding as these compounds are recorded to contain a diverse array of bioactivity from antivirals to hepatoprotectives [26,48]. There were no steroids predicted in the ethanol-based contents, likely due to extraction in different solvents. Extract degradation by oxidation was also confirmed as several hydroxide- and hydroperoxide-containing fatty acids attributed to a quenched polyunsaturated fatty acid called linoleic acid were found. Linoleic acid is known to be a medically beneficial and bioactive antioxidant metabolite; however, not much bioactivity was recorded for the oxidized forms of linoleic acids in the extracts except for biomarkers for atherosclerosis in humans [49].

Several predicted compounds in Figure 6a,b, highlighted in yellow, were found to be structurally unidentified when analyzed in various databases which may be a potential for new phytochemical compounds yet to be characterized or published. Despite this, chemical formulas of some unidentified compound highlighted in green were invalid as no carbons were detected. The invalid unnamed compounds were suspected to occur from overlaps of each potential hit of compound peaks, which were proven as there were some compounds that possessed large *m/z* values in the same run at around 800 *m/z* or above (Figure 6a).

While several of the valid unnamed compounds could not be analyzed further for details on classification and bioactivity, most of the known compounds were identified as bioactive, with many recorded as antioxidants and antibacterials (Table S1). Several examples known to have an overlap in strong antioxidant activity and potential for antibacterial activity against *S. aureus* or anti-MRSAs are the phenolic compounds aspirin and azelaic acid. Both compounds were reported to exhibit low anti-MRSA activity compared to antibiotics; however, when used together they synergistically support other antimicrobial and antibiotic compounds [50,51].

Other notable antioxidant compounds such as quercitrin and isovitexin were found to be able to inhibit an MRSA biofilm formation (Table S1). Both glycoflavonoids decrease MRSA virulence by inhibiting the Sortase A (SrtA) enzyme which anchors most Gram-positive peptidoglycan cell walls to other surface proteins. While isovitexin was able to reduce MRSA adhesion to fibrinogen as well as biofilm formation between colonies, it could not inhibit growth rate directly [52]. In silico and in vitro studies had also shown that quercitrin was able to bind to the enzymatic sites of wild-type as well as a mutant strain of *SrtA* gene-expressing colonies [53].

Due to the highly enriched composition of the extract as characterized by LC-MS/MS, the compounds inside the extracts will inadvertently interact with one another, resulting in either synergistic or antagonistic behavior [54]. Synergistic interactions can result in overall higher bioactivity, whereas antagonistic interactions can attenuate activities from certain compounds despite the overall high bioactivity. In this case, antioxidant activity was increased due to high levels of flavonoid- and tannin-based structures in the ethanol-based *S. caseolaris* leaves extract but not susceptible enough to inhibit MRSA due to its crudeness.

### 3.6. Relationship between Antioxidant and Antibacterial Activity from a Phytochemical Standpoint

**Figure 7:**
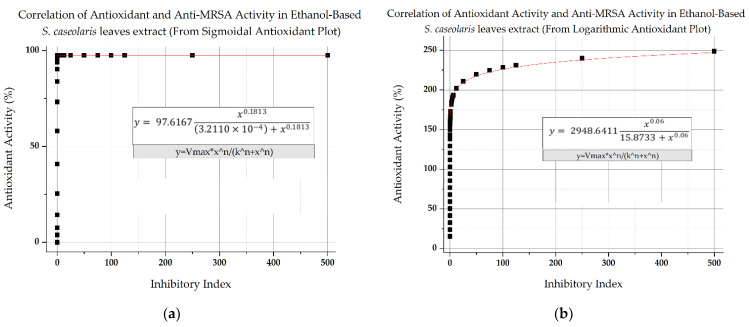
Correlation between antioxidant activity and antibacterial activity of ethanol-based *S. caseolaris* leaves crude extract against MRSA (**a**) using an antioxidant activity equation fitted in sigmoidal fitting and (**b**) in logarithmic fitting.

The antibacterial and antioxidant activity of the crude ethanol-based *S. caseolaris* leaves extract was plotted in single graphs using the resulting fitted equations with the extract concentration as their baseline. However, as there were two highly potential fittings (Supplementary Figures S1 and S2) for antioxidant activity, the correlations between different bioactivities were plotted in two separate figures where two antioxidant fitting equations were used (Figure 7a,b), with the overall correlation trend fitted using Hill’s equation sigmoidal fitting, with R^2^ reaching 0.8819 and 0.96612, respectively. Based on the figures, anti-MRSA would start to manifest at a concentration of 2000 ppm with an inhibitory index of 0.001 in crude extract, similar to the faint indication in Figure 3a. At 2000 ppm, the hypothetical antioxidant activity would have already reached almost maximum DPPH radical scavenging inhibition at 97.5% using sigmoidal fitting (Figure 7a), whereas activity would surpass 100% at 141% using logarithmic fitting (Figure 7b). In regard to Vancomycin, the extract would start to surpass the antibiotic at 62,000 ppm with a theoretical inhibitory index of 3.001, while antioxidant activity would be approximately 97.6% and 182%, according to the respective antioxidant fittings.

The antioxidant activity of the ethanol-based *S. caseolaris* leaves extract was predicted to reach maximum DPPH inhibition capacity based on the initial antioxidant sigmoidal fitting (Figure 7a and Figure S1), likely due to the high phenolic and flavonoid contents initially labeled as super-antioxidants. In the antioxidant determination experiment, the amount of DPPH radicals was limited within the closed system, which contributed to inhibition stagnation. By following this correlation fitting, antioxidative phytochemicals in crude extracts are suggested to exhibit anti-MRSA activity long after oxidative stresses in a closed system are fully quenched due to attenuation of antibacterial activity. This might become a problem as antioxidants are more likely to turn into prooxidants when used in larger doses, hypothesized to be related to how quenched antioxidants replenish their lost protons [55,56].

Another correlation fitting based on an open system was proposed as an alternative, using antioxidant activity fitted in logarithmic fitting (Figure 7b and Figure S2), to visualize the correlation of both activities if the model has a non-exhaustive source of radicals. Based on Figure 7b, it was also suggested that there is a potential hit for enzymatic-based activity and mode of action for both bioactivities, albeit still in a very inefficient manner similar to Figure 7a, due to the large concentrations needed to show first signs of antibacterial activity. The low antibacterial activity seemed to be associated with the competition inhibitors-like interactions between major compounds with enzymes found within MRSA, causing higher cases of activity attenuation. While synergistic and additive effects of phytochemicals are more frequent in nature, antagonistic effects can happen due to inactive compounds and other metabolites working as a growth substrate for the bacteria [57]. It is noted that while further studies should be taken immediately to determine the structure-assisted relationship (SAR) of each anti-MRSA and antioxidant activity contributors, there is an overall correlation between biological activities for the major compounds which took the form of a first-order equation.

While the underlying mechanism that ties both antioxidant and antibacterial activity has long been debated, most phytochemicals attributed to antioxidant activity have been documented to also be a contributor for antibacterial activity. One hypothesis stemmed from how both phytochemicals for antioxidant and antibacterial activity perform similar electron transfer reaction mechanisms [55,58]. Removal or reduction of ROS and radical nitrogen species (RNS) in the environment could be related to antibacterial activity, due to how most aerobic microbes need oxygen or nitrogen-based compounds for their survival [56,59]. Crude methanolic extracts of the same species were able to reduce non-pathogenic microbial growths while were also reported to contain multiple antioxidative phytochemical groups such as triterpenes and kaempferol, though it was also speculated that the overall bioactivity was caused by synergistic activity from other chemicals [45]. Based on Figure 7a,b, it seemed that, indeed, both antioxidant and antibacterial activity showed positive correlation, with antioxidant activity predicted to greatly increase anti-MRSA activity, as suggested by the ROS deprivation hypothesis. However, this predicted correlation needs further testing, as MRSA strains are facultative anaerobic bacteria, meaning that they could live both aerobically or anaerobically, depending on the stress condition [60].

In terms of mechanism of action, antibacterial activity-exhibiting phytochemicals may produce antioxidant activity as an additive from their active aromatic structures and scavenging-capable moieties [9,36]. Several antioxidative flavonoids were documented to alter the membrane fluidity of certain types of bacteria [61]. Glucosides flavonoids were believed to be more stable, soluble, and increase the bioavailability of flavonoids [26,48,62]. One example of glycosylation of flavonoids is the conversion of quercetin to quercitrin, both of which were antitrypanosomal and antileishmanial potentials [63]. Other phenolic compounds such as tannins are also known to be multiple metabolic pathway suppressors and enzyme inhibitors, either for efflux pumps on MDR strains or enzymes in DNA replication [59,64]. Several potent flavonoid compounds were able to strongly inhibit different MRSA strains, with several of them identified to be able to inhibit enzymes associated with DNA-RNA synthesis [65]. Phytochemical characterization of the ethanol-based *S. caseolaris* leaves crude extract revealed the presence of enzyme-inhibiting antioxidants (isovitexin and quercitrin), potential hits of enzymatic activity (correlation interpretation in Figure 7a,b), as well as synergistic activity-exhibiting compounds (aspirin and azelaic acid). However, fractionation to more purified compounds and further tests on the MIC is critical to help identify the source of additive and synergistic interactions for both bioactivities.

## 4. Conclusions

Water-based *A. marina*, water-based *R. mucronata*, water-based *R. apiculata*, and ethanol-based *S. caseolaris* leaves crude extracts taken from the locale were characterized for bioactive phytochemical compounds and assayed to evaluate antibacterial activity against the MRSA strain MDR-*S. aureus*. *S. caseolaris* leaves extract was found to be non-lethal to *A. salina* in a lethality assay and was even found to increase viability, which was backed culturally by how the mangrove species are used as a staple food. It was also found that the nontoxic *S. caseolaris* leaves extract contained the highest yield of phenolic phytochemicals, among which were identified as flavonoid glycosides and other phenolic glycosides using LC-MS/MS. The crude extract was also found to exhibit the strongest DPPH antioxidant activity, with the DPPH IC_50_ being highly comparable to ascorbic acid, to the point that the extract and plant part of species *S. caseolaris* leaves could be deemed as a source of super-antioxidants.

In terms of antibacterial activity, the only actively inhibiting extracts observed were from the ethanol solvent-based *S. caseolaris* leaves extract. MRSA growth was inhibited around the crude extract starting from 5000 ppm diluted in ethanol solvent, with ZOI diameter reaching up to 14mm. However, the extract was highly crude in composition and did not surpass the threshold for a susceptible or intermediate level for antibacterial activity against the control drug Vancomycin as stated in EUCAST 2022 MIC breakpoint table. Preliminary correlation prediction on antibacterial and antioxidant activity showed promising results in potential hits for enzymatic-based mechanism and synergistic activity despite the crudeness of the extract. Nevertheless, sample extracts can still be deemed as a candidate for anti-MRSA. Further studies on metabolomics and fractionation are very much needed in order to elevate the validity of high super-antioxidant and anti-MRSA activity potential.

## Figures and Tables

**Table 1:** Phytochemical screening for dried simplicial leaves of various local mangrove species leaves, as performed by Audah et al. [16] as a preliminary study.

Sample	Alkaloid	Triterpenoid	Steroid	Quinone	Flavonoid	Saponin	Tannin
*Sonneratia caseolaris* leaves	-	-	+	-	++	-	+++
*Avicennia marina* leaves	-	-	+	-	++	++	++
*Rhizophora mucronata* leaves	-	-	++	-	+	+++	+++
*Rhizophora apiculata* leaves	-	-	++	-	-	+	++

**Table 2:** Preliminary tests of various local mangrove species leaves extracts for solvent optimization using mass yield percentage and toxicity to *A. salina* as performed by Audah et al. [16].

Plant Species and Parts	Types of Solvent	Mass Yield Percentage (%)	LC_50_ (ppm) on *A. salina*
*S. caseolaris* leaves	N-hexane	1.517 ± 0.254	633.59
	Ethyl Acetate	1.431 ± 0.089	546.08
	Ethanol	9.464 ± 0.096	1076.05
	Water	9.380 ± 4.208	488.93
*A. marina* leaves	N-hexane	1.829 ± 0.676	878.62
	Ethyl Acetate	1.398 ± 0.072	1254.44
	Ethanol	7.422 ± 1.171	229.77
	Water	26.227 ± 5.130	847.63
*R. mucronata* leaves	N-hexane	1.036 ± 0.133	160.43
	Ethyl Acetate	2.358 ± 0.741	224.45
	Ethanol	2.485 ± 0.511	498.28
	Water	13.263 ± 3.745	772.70
*R. apiculata* leaves	N-hexane	1.281 ± 0.381	734.36
	Ethyl Acetate	3.220 ± 2.090	918.78
	Ethanol	6.546 ± 3.695	722.40
	Water	16.196 ± 5.198	627.48

## Data Availability

Publicly available datasets were analyzed in this study. This data can be found here: [https://drive.google.com/drive/folders/1LERr0ivJcVvdhoizQfhPVLH6aaG-tHzM?usp=sharing] (accessed on 14 September 2022). Supplementary datasets are also available on request from the corresponding author.

## Supplementary Materials

The following supporting information can be downloaded at: https://www.mdpi.com/article/10.3390/molecules27238369/s1, Figure S1: Antioxidant activity of ethanol-based *S. caseolaris* leaves extract using sigmoidal fitting, Figure S2: Antioxidant activity ethanol-based *S. caseolaris* leaves extract using logarithmic fitting, Table S1: Analysis on structure of various phytochemical compounds and respective documented bioactivity found in the ethanol-based solvent *S. caseolaris* leaves extract, with interested compounds highlighted in yellow along with known bioactivity related to Antioxidant and Anti-MRSA, Supplementary Materials Dataset Excel sheet, Supplementary Dataset for Antioxidant OriginPro sheet, and Supplementary Dataset for Correlation OriginPro sheet. References [66,67,68,69,70,71,72,73,74,75,76,77,78,79,80,81,82,83,84,85,86,87,88,89] are cited in the Supplementary Materials.

## Sample Availability

All four plant extract samples used in this study are available from the corresponding author.
