# Supplementary material for: Indonesian Mangrove Sonneratia caseolaris Leaves Ethanol Extract Is a Potential Super Antioxidant and Anti Methicillin-Resistant Staphylococcus aureus Drug
Source: Molecules. 2022 Nov 30;27(23):8369. doi: 10.3390/molecules27238369 (PMC9735687; doi:10.3390/molecules27238369)
Supplement: Supplementary file 1 [file molecules-27-08369-s001.zip › Supplementary Material File.pdf]

Indonesian Mangrove *Sonneratia caseolaris* Leaves Ethanol Extract is a Potential Super Antioxidant and Anti Methicillin-Resistant *Staphylococcus aureus* Drug

Kholis Abdurachim Audah[1,2,*], Jufendi Ettin[1], Jason Darmadi[1] , Norma Nur Azizah[3], Amalda Siti Anisa[1], Tedi Dwi Fauzi Hermawan[1], Conny Riana Tjampakasari[4], Rudi Heryanto[5,6], Intan Safinar Ismail[7] and Irmanida Batubara[5,6]

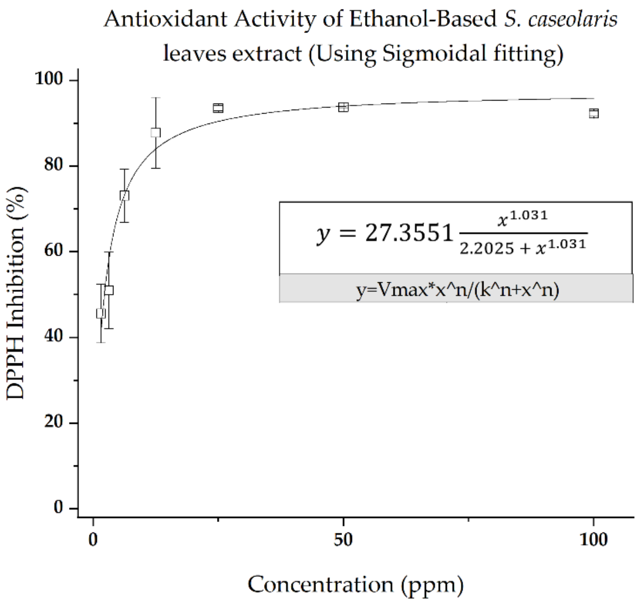

Figure S1. Antioxidant activity of ethanol-based *S. caseolaris* leaves extract using sigmoidal fitting

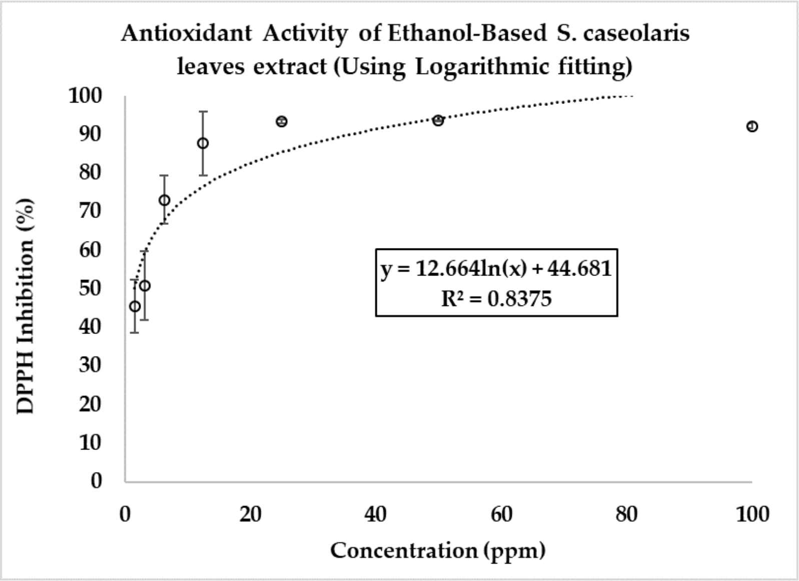

Figure S2. Antioxidant activity ethanol-based *S. caseolaris* leaves extract using logarithmic fitting.

Table S1. Analysis on structure of various phytochemical compounds and respective documented bioactivity found in the ethanol-based solvent *S. caseolaris* leaves extract, with interested compounds highlighted in yellow along with known bioactivity related to Antioxidant and Anti-MRSA

No.	Chemical Formula and Structure	Compound Name	Metabolite Classification	Specific Phytochemical Type	Known Bioactivity	References
1	$C_5H_{12}O_6$ 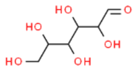	Hexose	Sugar	-	Not available	-

2	$C_6H_{14}O_6$ 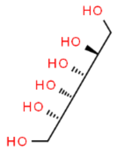	Sorbitol	Sugar alcohol	-	Laxative effects, antioxidant, antifungal	[66-68]
3	$C_{15}H_{21}O_{10}$	Methoxy [2,3,4,5 tetrakis (methocarbonyl) cyclopentyl] methanolate	Not available	Not available	Not available	-
4	$C_9H_{15}O_4$ 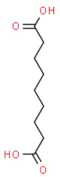	Azelaic acid	Phenolic compound	Phenolic acid (dicarboxylic acid)	Anti-inflammatory, antioxidant, antimicrobial, low anti-MRSA, antiacne, anti-*Staphylococcus aureus*	[50,69-71]
5	$C_9H_7O_4$ 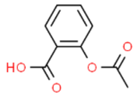	Aspirin / 2-acetoxybenzoic acid	Phenolic compound	Phenolic acid (Salicylic acid)	Anti-inflammatory, antioxidant, antimicrobial, anti-*S. aureus*, antithrombosis, analgesic, antipyretic, low anti-MRSA	[51,72,73]
6	$C_7H_5O_5$ 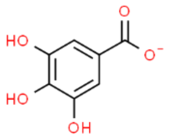	Gallate / 3,4,5-trihydroxybenzoate	Phenolic compound	Phenolic acid (gallic acid)	Antioxidant, anticancer, anti-inflammatory, antimicrobial, antiviral, antiallergen, anti-*S. aureus*, prooxidant	[74,75]
7	$C_{25}H_{28}O_{16}$ 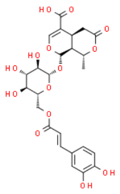	Ibotalactone B	Phenolic compound	Phenolic glycosides (Secoiridoid)	Not available	-

8	$C_{16}H_{22}O_8$ 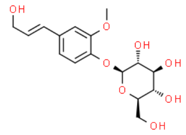	Abietin / Coniferin	Phenolic compound	Phenolic glycosides	Antioxidant, prooxidant, antidiabetic	[76-78]
9	$C_{20}H_{30}O_{10}$ 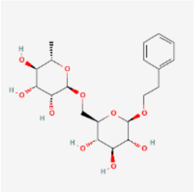	2-Phenylethyl 6-O-(6-deoxy-α-L-mannopyranosyl)-β-D-glucopyranoside / 2-Phenylethyl D-rutinoside	Phenolic compound	Phenolic glycosides	Anti-inflammatory, antioxidant, nephroprotective	[79]
10	$C_{27}H_{29}O_{15}$ 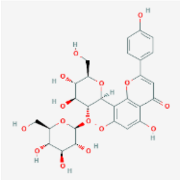	Vitexin 2-O-β-D-glucoside / flavosativaside	Flavonoids	Flavone glycosides	Not available	[80]
11	$C_{21}H_{20}O_{10}$ 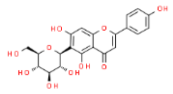	Isovitexin	Flavonoids	Flavone glycosides	Antioxidant, anti-MRSA biofilm adhesion / virulence, antimicrobial	[52,81-83]
12	$C_{21}H_{19}O_{11}$ 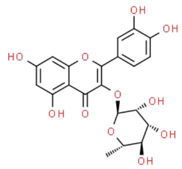	Quercitrin	Flavonoids	Flavonol glycosides	Anti-inflammation, antioxidant, anti-MRSA biofilm adhesion / virulence, low antimicrobial, low anti-*S. aureus*	[53,84,85]

13	$C_{15}H_9O_6$ 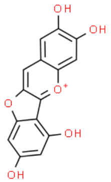	Riccionidin A	Flavonoids	Anthocyanin glycosides	Suspected antimicrobial and antichromists, antioxidant	[86-88]
14	$C_{26}H_{27}O_{15}$ 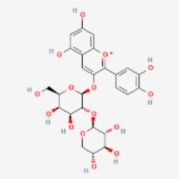	Cyanidin 3-O-[β-D-xylosyl-(1-2)- β-D-galactoside]	Flavonoids	Anthocyanin glycosides	Not available	-
15	$C_{14}H_5O_8$	3,7,8,-trihydroxy-5,10-dioxo-5,10-dihydrochromeno[5,4,3-cde]chromen-2-olate	Coumarins	-	Not available	-
16	$C_{18}H_{32}O_3$ 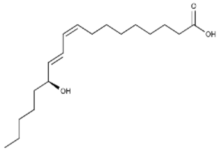	13S-hydroxyoctadecadienoic acid / 13S-HODE	Long-chain fatty acid	Fatty acyls	Anticancer, prooxidant	[89]
17	$C_{18}H_{31}O_5$	9-Hydroperoxy-11-(3-pentyl-2-oxiranyl)-10-undecenoate	Long-chain fatty acid	Fatty acyls	Not available	-
18	$C_{18}H_{33}O_5$ 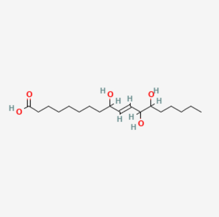	9,12,13-trihydroxy-10-octadecenoate / 9,12,13-TriHOME(10)	Long-chain fatty acid	Fatty acyls	Not available	-
19	$H_3O_{12}NClP_{10}S_9$	Unnamed*	-	-	-	-

20	$H3O_{11}NClP_{10}S_9$	Unnamed*	-	-	-	-
21	$H_4O_6N3Cl_2P_{10}S_9$	Unnamed*	-	-	-	-
22	$H_3O_{10}NClP_{10}S_9$	Unnamed*	-	-	-	-
23	$C_{30}H_{51}O_5N_{10}S$	Unnamed	Not available	Not available	Not available	-
24	$C_{30}H_{51}O_4N_{10}S$	Unnamed	Not available	Not available	Not available	-
25	$C_{12}H_{33}ON_2P_8$	Unnamed	Not available	Not available	Not available	-

* Invalid results due to no carbon elements detected
